# Hypoxic vasodilatory defect and pulmonary hypertension in mice lacking hemoglobin **β**-cysteine93 *S*-nitrosylation

**DOI:** 10.1172/jci.insight.155234

**Published:** 2022-02-08

**Authors:** Rongli Zhang, Alfred Hausladen, Zhaoxia Qian, Xudong Liao, Richard T. Premont, Jonathan S. Stamler

**Affiliations:** 1 Institute for Transformative Molecular Medicine, Department of Medicine and; 2 Cardiovascular Research Institute, Case Western Reserve University School of Medicine, Cleveland, Ohio, USA.; 3 Harrington Discovery Institute, University Hospitals Cleveland Medical Center, Cleveland, Ohio, USA.

**Keywords:** Cardiology, Vascular Biology, Hypoxia, Microcirculation, Nitric oxide

## Abstract

Systemic hypoxia is characterized by peripheral vasodilation and pulmonary vasoconstriction. However, the system-wide mechanism for signaling hypoxia remains unknown. Accumulating evidence suggests that hemoglobin (Hb) in RBCs may serve as an O_2_ sensor and O_2_-responsive NO signal transducer to regulate systemic and pulmonary vascular tone, but this remains unexamined at the integrated system level. One residue invariant in mammalian Hbs, β-globin cysteine93 (βCys93), carries NO as vasorelaxant *S*-nitrosothiol (SNO) to autoregulate blood flow during O_2_ delivery. βCys93Ala mutant mice thus exhibit systemic hypoxia despite transporting O_2_ normally. Here, we show that βCys93Ala mutant mice had reduced *S*-nitrosohemoglobin (SNO-Hb) at baseline and upon targeted SNO repletion and that hypoxic vasodilation by RBCs was impaired in vitro and in vivo, recapitulating hypoxic pathophysiology. Notably, βCys93Ala mutant mice showed marked impairment of hypoxic peripheral vasodilation and developed signs of pulmonary hypertension with age. Mutant mice also died prematurely with cor pulmonale (pulmonary hypertension with right ventricular dysfunction) when living under low O_2_. Altogether, we identify a major role for RBC SNO in clinically relevant vasodilatory responses attributed previously to endothelial NO. We conclude that SNO-Hb transduces the integrated, system-wide response to hypoxia in the mammalian respiratory cycle, expanding a core physiological principle.

## Introduction

The physiological response to systemic hypoxia is a foundational aspect of the respiratory cycle through which O_2_ is delivered to tissues. Systemic blood vessels dilate and pulmonary vessels constrict under hypoxia to improve O_2_ delivery to tissues (1). However, the integrated molecular mechanisms for O_2_ sensing are not well understood. Accumulating evidence suggests (i) that the respiratory cycle is in fact a 3-gas system in which hemoglobin (Hb) is a carrier for not just 2, but rather 3 gasses in blood: O_2_, CO_2_, and NO (2, 3) and (ii) that Hb acts as an O_2_-responsive, NO-based vasodilator that matches tissue perfusion to O_2_ demand (4, 5). This effect, termed autoregulation of blood flow (5), acts locally within individual capillaries and microvascular beds to increase RBC transit and functions in direct proportion to Hb desaturation to ensure metabolic coupling (together with vasodilators released from hypoxic tissues for capillary recruitment; ref. 6). Mice mutated to be unable to dispense NO from Hb are therefore profoundly hypoxic despite RBCs carrying normal amounts of O_2_ (7).

Although both O_2_ and NO bind to hemes in Hb, NO can also react with Hb’s sulfhydryl groups to form *S*-nitrosothiols (SNOs; refs. 8, 9). In the form of SNO, NO bioactivity is preserved in Hb (whereas NO bound to heme is inactive), and SNO on proteins can act as a signaling modification to regulate protein allostery (8). Conversion of NO to SNO in Hb takes place within the β-globin subunit (9). It has been shown that NO binding to β-globin heme, particularly Fe^3+^ heme, serves to redox-activate NO to NO^+^ (nitrosonium ion) that can then *S*-nitrosylate a conserved β-globin cysteine93 (βCys93) to form βCys93-SNO (10, 11); Hb thus acts as an SNO synthase (12). Also, it has been shown that the reaction of NO with βCys93 is coupled to the allosteric transition in Hb (13), with SNO formation favored in the oxygenated/R state of Hb and SNO release favored in the deoxygenated/T state. SNO-Hb thus serves as an O_2_-responsive NO buffer, only releasing SNO in the hypoxic T state (14, 15). In short, when Hb releases O_2_ in hypoxic tissues, it undergoes a shift from R to T conformation, causing βCys93 to transfer NO^+^ to other thiols that transport NO out of RBCs to increase blood flow (15). Thus, Hb-derived SNO has been proposed as the mediator of classical autoregulation of tissue blood flow that is proportional to Hb O_2_ saturation (5).

This model of SNO-Hb–mediated hypoxic vasorelaxation and O_2_ delivery has now been supported by numerous studies, both in vitro and in vivo, including fairly dispositive genetic evidence. These data include demonstration that isolated human Hb but not Cys93Ala (C93A) mutant Hb (16) can be *S*-nitrosylated by physiological amounts of NO (12), that this SNO-Hb is vasodilatory but only under hypoxia (13, 15), that the vasorelaxant activity of native RBCs and of physiological amounts of SNO in RBCs requires hypoxia (13, 17, 18), and that autoregulation of blood flow and tissue oxygenation in vivo are profoundly disrupted by mutation in βCys93 (7). Further, we and others have demonstrated profound hypoxia-related phenotypes in these mice, including deficiencies in tissue oxygenation and flow-mediated vasodilation, increased development of coronary collateral vessels, cardiac dysfunction, altered breathing response to hypoxia, and death upon acute exposure to a very low O_2_ environment (7, 19, 20). Nevertheless, one group of authors have disputed this model, specifically by failing to observe hypoxia-dependent vasodilation from SNO-loaded RBCs in vitro (21, 22) and reporting that mice with βCys93 mutation exhibit no significant phenotypes as tested, including absence of pulmonary hypertension (18, 21–25).

In this regard, systemic hypoxia results in sine qua non changes in peripheral vascular tone and contrasting changes in pulmonary vascular tone: i.e., hypoxia induces vasodilation in peripheral microvasculature and instead vasoconstriction in pulmonary microvasculature to promote ventilation/perfusion matching (26). Notably, the molecular mechanism for O_2_ sensing at an integrated system level remains a major unanswered question in vascular physiology, and thus the extent to which RBCs may contribute is unknown. While C93A mice would seem to be the ideal model to test this question, mouse RBCs differ from human RBCs in terms of vasodilatory mechanisms (25, 27) and have not been previously optimized for SNO formation, by contrast with human RBCs (15), thus limiting interpretations in vitro (25, 28). In particular, current methods to load SNOs in mouse RBCs (20, 21) produce very high levels of met-Hb (oxidized Hb that cannot carry O_2_) and non-Hb SNOs, which mask C93 activity. Here, we develop tests of fundamental RBC physiology in vitro and in vivo to reveal the role of βCys93 in peripheral and pulmonary vascular responses. Our results indicate that RBCs serve in system-wide O_2_ sensing and O_2_-responsive SNO signaling, to regulate systemic and pulmonary vascular tone, expanding a core principle in physiology.

## Results

### Hb βCys93 mediates hypoxic vasodilation in vitro.

**Figure 1:**
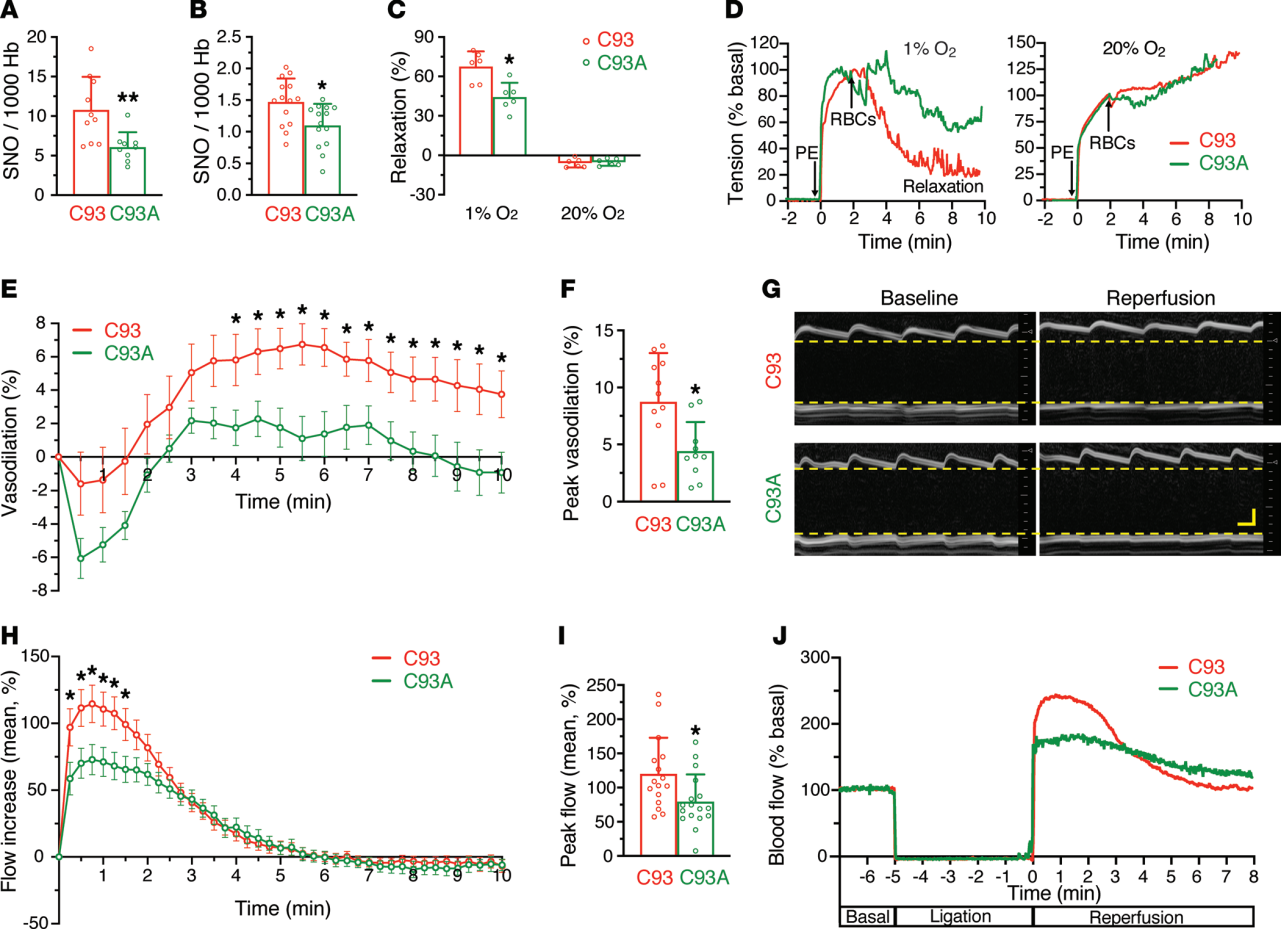
Reduction of SNO-Hb in Cys93A RBCs and impairment of hypoxic vasodilation. (**A**) SNO levels after treatment of RBCs from C93 control and C93A mice with CysNO (*n* = 10 for C93; *n* = 9 for C93A). (**B**) Baseline SNO in untreated RBCs from C93 and C93A mice (*n* = 14 each). (**C**) RBC-mediated hypoxic vasodilation of isolated aortic rings in vitro. SNO-loaded RBCs in **A** were added to bioassays under hypoxia (1% O_2_) or normoxia (20% O_2_) (*n* = 6 each). Data shown as mean ± SD. *P < 0.05, **P < 0.01 vs. C93, 2-tailed Student’s *t* test. (**D**) Representative aortic ring bioassay response to adding SNO-loaded C93 versus C93A RBCs (arrow) over time, in 1% O_2_ or 20% O_2_, normalized to 100% tension with phenylephrine. (**E**) Vasodilation in abdominal aorta in vivo at baseline and after aortic ligature release. Data shown as mean ± SEM. *n* = 11 C93 (4.0 ± 0.3 months); *n* = 10 C93A (3.7 ± 0.4 months). *P < 0.05 vs. C93, 2-way ANOVA. (**F**) Peak vasodilation of aorta, calculated from peak response from each mouse. Data shown as mean ± SD. *P < 0.05 vs. C93, 2-tailed Student’s *t* test. (**G**) Representative short axis M-mode images of abdominal aorta depicting aortic dilation (and impairment in C93A). Dashed lines represent vessel wall positions at diastole, for calculating diameter. Vertical scale bar: 2 mm; horizontal: 50 ms. (**H**) Mean blood flow increase after aortic ligature release. Data shown as mean ± SEM. *n* = 16 C93 (3.8 ± 0.9 months); *n* = 17 C93A (3.8 ± 0.7 months). *P < 0.05 vs. C93, 2-way ANOVA. (**I**) Peak flow increase, calculated from each mouse. Data shown as mean ± SD. *P < 0.05 vs. C93, 2-tailed Student’s *t* test. (**J**) Representative abdominal aortic blood flow curves.

We have previously shown that native βCys93 mutant RBCs induce vasodilation less effectively under hypoxia than control RBCs (7) and that human RBCs loaded physiologically with NO gas recapitulate hypoxic vasodilation by native RBCs (15). However, it has been reported recently that mouse RBCs preloaded with SNO (via treatment with CysNO) do not show hypoxic vasodilation or differences in vasodilation between mutant and control RBCs (22). In working with mouse RBCs, we noted that Hb was not modified by exogenous CysNO as readily as was Hb within human RBCs and that met-Hb (which eliminates the allosteric transition in Hb) formed in very high amounts (data not shown). We therefore developed a protocol (see Methods) optimized for SNO loading of Hb within humanized mouse RBCs. With this procedure, we are able to load SNO predominantly onto βCys93, although mouse RBCs load SNO less well and produce higher met-Hb than do normal human RBCs. Under these optimized loading conditions, the C93 (control) mouse RBC preparations contained approximately 10 SNO per 1000 Hb tetramers (~2 SNO/1000 heme) and were oxidized to approximately 10% met-Hb, while C93A (mutant) RBCs had significantly less SNO (~6 SNO per 1000 Hb tetramers or ~1 SNO/1000 heme) (Figure 1A) and indistinguishable met-Hb levels. CysNO increased SNO-Hb 5-fold over the level observed in fresh, untreated C93 RBCs and in C93A mutant RBCs, which had significantly less SNO-Hb also at baseline (Figure 1B). Adding these SNO-loaded C93 RBCs to aortic ring bioassays from wild-type mice resulted in vasorelaxation under hypoxia, but not under normoxia (Figure 1, C and D) (as seen with physiological amounts of SNO-Hb and native RBCs; refs. 25, 29), fulfilling the sine qua non requirement of hypoxic vasodilation. In contrast, SNO-loaded C93A RBCs produced significantly less vasorelaxation under hypoxic conditions than C93 RBCs but were equally vasoconstrictive under normoxia. These results demonstrate that Hb βCys93 is the primary and preferred site of *S*-nitrosylation in RBCs, that carefully loaded mouse RBCs recapitulate the hypoxic vasorelaxation found using human RBCs under basal conditions (14, 15, 25), and that this hypoxic vasorelaxation effect is significantly diminished when SNO can no longer bind to or be released from βCys93. Thus, the allosterically regulated βCys93 mediates hypoxic vasodilation by RBCs.

### Peripheral vasodilation by Hb βCys93 in vivo.

In vitro bioassays with isolated aortic rings and static, dilute RBCs have limitations. To assess the role of Hb βCys93 in regulating hypoxic vasodilation in vivo, we performed 2 types of experiments based on classic reactive hyperemia paradigms (30, 31) but using abdominal aorta in situ. First, flow through the abdominal aorta was blocked temporarily by ligating the abdominal aorta for 5 minutes to create tissue hypoxia, the ligature was released, and the diameter of the abdominal aorta upstream of the ligation site was measured at diastole in real time using ultrasonography. In this model of reactive hyperemia, the diameter of the aorta underwent a transient increase after hyperemic flow had normalized, and the extent of this increase, shown as dynamic vasodilation, was significantly reduced in the C93A mice (Figure 1E). The overall dilation of the abdominal aorta increased by approximately 8.8% over the basal diameter in C93 (control) mice bearing wild-type human Hb (Figure 1F) compared with the diameter at the same location prior to occlusion. By contrast, the diameter increase in C93A mouse aorta was significantly blunted, at only approximately 4.4% over its basal diameter (Figure 1F); representative M-mode images for individual mice are shown in Figure 1G. This indicates that Hb βCys93-derived SNO within RBCs contributes about 50% of the vasodilation effect following temporary occlusion. The remaining half is attributed to local, shear force–induced endothelial NO production upon restored flow (flow-mediated dilation, FMD; refs. 32, 33), which has often been assumed to be responsible for the full effect but without empirical evidence. Moreover, it had not been previously possible to discern the role of endothelium versus RBCs because endothelial NOS (eNOS) inhibition reduces levels of SNO-Hb and RBC SNO (13, 34). We conclude that vasodilation following occlusion is evidently mediated by both RBCs and endothelium, the former stimulated by hypoxia (35) and the latter by shear.

In a second experiment, we directly measured blood flow through the abdominal aorta downstream of the ligation site using an ultrasound flow probe. Increases in flow following ligature release result from microcirculatory vasodilation downstream, which provides a surrogate measure of NO vasodilatory activity. Generally, very small diameter increases distributed across the microcirculation result in marked increases in flow, as flow is a function of (radius)^4^. As with abdominal aorta diameter, we measured blood flow continually through the cardiac cycle and calculated systolic peak and mean blood flow for the first second of each 15-second window to determine dynamic flow increases. Mean flow increase is shown in Figure 1H. In C93 control mice, the mean aortic blood flow increased nearly 1.2-fold over flow measured prior to the blockade, but in the C93A mice, this flow was significantly blunted, reaching only approximately 0.8-fold (Figure 1I); representative traces for individual mice are shown in Figure 1J. Similarly, peak flow at systole was increased 54% over basal in control mice, but this was significantly reduced to 31% in C93A mice (Supplemental Figure 1, A and B; supplemental material available online with this article; https://doi.org/10.1172/jci.insight.155234DS1). We conclude that about 50% of the hyperemic flow increase, reflecting microcirculatory vasodilation following hypoxia, is mediated by Hb βCys93 within RBCs.

### Right ventricular hypertrophy in young and aged animals in the absence of βCys93.

**Figure 2:**
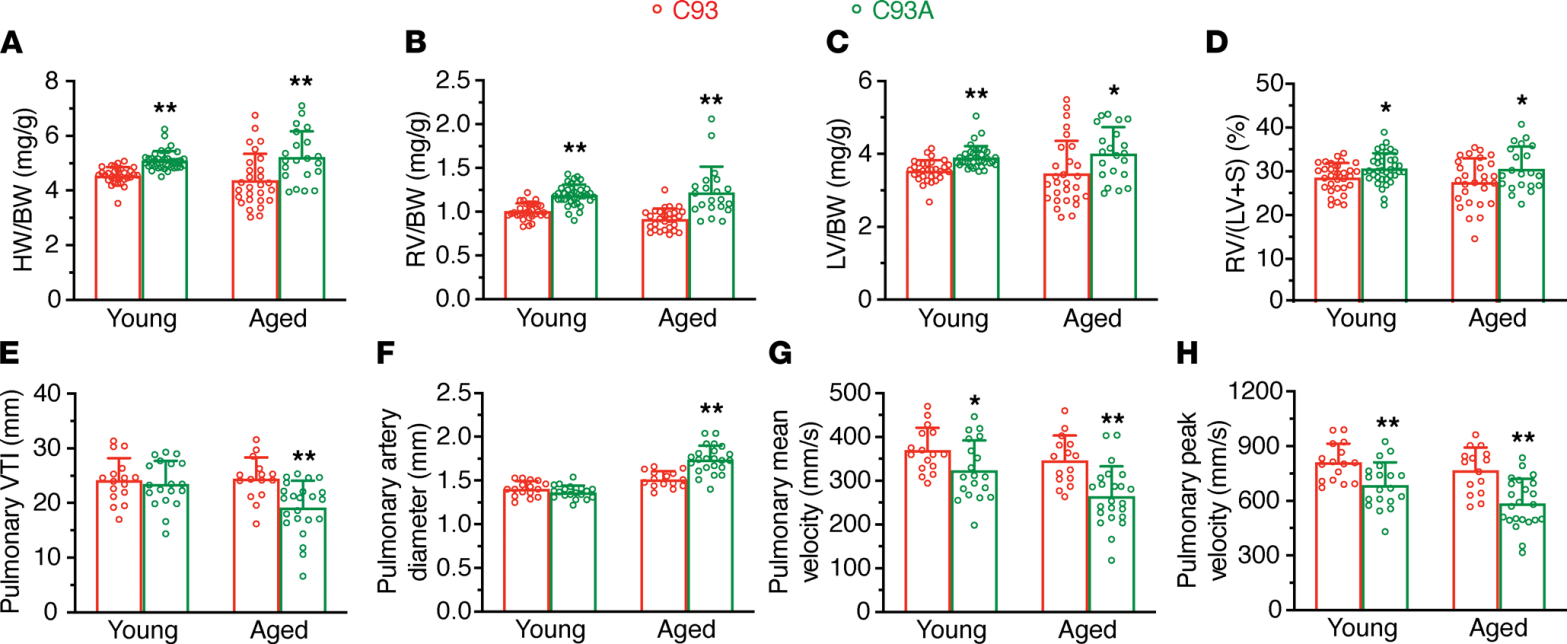
Right ventricular and pulmonary artery signs of pulmonary arterial hypertension in C93A mice with age. (**A**) Total heart weight (HW) to body weight (BW) ratio in young and in aged C93A versus C93 mice. (**B**) Right ventricle (RV) to BW ratio in young and in aged C93A versus C93 mice. (**C**) Left ventricle (LV) to BW ratio in young and in aged C93A versus C93 mice. (**D**) RV to LV + septum weight (LV+S) ratio in young and in aged C93A versus C93 mice. (**E**) Pulmonary artery (PA) blood flow velocity-time integral (VTI) in young and in aged C93A versus C93 mice. (**F**) PA diameter in young and in aged C93A versus C93 mice. (**G**) Mean velocity of PA blood flow in young and in aged C93A versus C93 mice. (**H**) Peak velocity of PA blood flow in young and in aged C93A versus C93 mice. For panels **A**–**D**, young mice (*n* = 31 C93, 3.4 ± 0.6 months of age, and *n* = 36 C93A, 3.4 ± 0.4 months of age) and aged mice (*n* = 28 C93, 19.5 ± 1.8 months of age, and *n* = 21 C93A, 19.7 ± 3.1 months of age) were assessed. For panels **E**–**H**, young mice (*n* = 16 C93, 3.8 ± 1.3 months of age, and *n* = 19 C93A, 3.0 ± 0.8 months of age) and aged mice (*n* = 15 C93, 20.9 ± 1.6 months of age, and *n* = 23 C93A, 21.8 ± 1.2 months of age) were assessed. Differences were assessed using Student’s *t* test (2 tailed). *P < 0.05, **P < 0.01 C93A vs. C93, for young or aged animals compared separately.

Loss of ability to carry and release SNO from Hb βCys93 leads to tissue hypoxia that is compensated in part by increased cardiac workload (7). Additionally, hypoxia may result in pulmonary hypertension, evidenced by right ventricular hypertrophy. We found total heart weight to body weight ratio to be significantly elevated in both young adult and old C93A mice, compared with C93 control mice (Figure 2A). Further, both right ventricle weight (Figure 2B) and left ventricle weight (Figure 2C) as a ratio to body weight were elevated significantly in C93A mice. However, C93A mice were lighter than C93 controls, sufficiently so to potentially confound interpretation when aged (C93 26.86 ± 2.76 g and C93A 25.04 ± 2.00 g for young mice, *P* < 0.01; and C93 42.82 ± 7.91 g and C93A 34.23 ± 4.43 g for aged mice, *P* < 0.01). More importantly, the ratio of right ventricle weight to the weight of the left ventricle plus septum (Figure 2D) was significantly elevated in both young and aged C93A mice (independent of body weight differences). Thus, both young and aged C93A mice showed signs of right heart hypertrophy, suggesting increased pulmonary vascular tone.

### Age-related signs of pulmonary hypertension in the absence of βCys93.

While young adult C93A mice had normal pulmonary artery blood flow velocity-time integral (VTI), pulmonary VTI was significantly reduced in aged C93A mice (Figure 2E). Likewise, pulmonary artery diameter was normal in young C93A mice but was significantly increased in aged C93A mice (Figure 2F). Mean and peak blood velocity in the pulmonary artery were both significantly reduced in both young and aged C93A mice (Figure 2, G and H).

### Pulmonary hemodynamics in young and aged animals.

In contrast to the prior (7) and above data in conscious mice, in young anesthetized mice at baseline (Supplemental Table 1), no significant changes were apparent, while in aged anesthetized C93A mice, systolic, diastolic, and mean pulmonary arterial pressures and right ventricular systolic pressure trended toward increases versus control mice (Supplemental Figure 2, A–D). Measures of right ventricular function in aged versus young mice, including *dP/dt* max, *dP/dt* min, contractility index, and average *dP/dt* over the isovolumic relaxation period (IRP average *dP/dt*) showed a similar trend (Supplemental Figure 2, E–H). Taken together with right ventricular hypertrophy and pulmonary artery dilation in aged C93A animals at baseline, and age-related reductions in pulmonary blood flow velocity in conscious C93A mice (Figure 2), our results suggest that with activity, stress, and aging, abnormal pulmonary vascular reactivity and/or right ventriculo-arterial coupling results in right-sided dysfunction at baseline, while in unstressed animals under anesthesia, right-sided changes are attenuated.

### Pulmonary hypertension with chronic hypoxia in the absence of βCys93.

**Figure 3:**
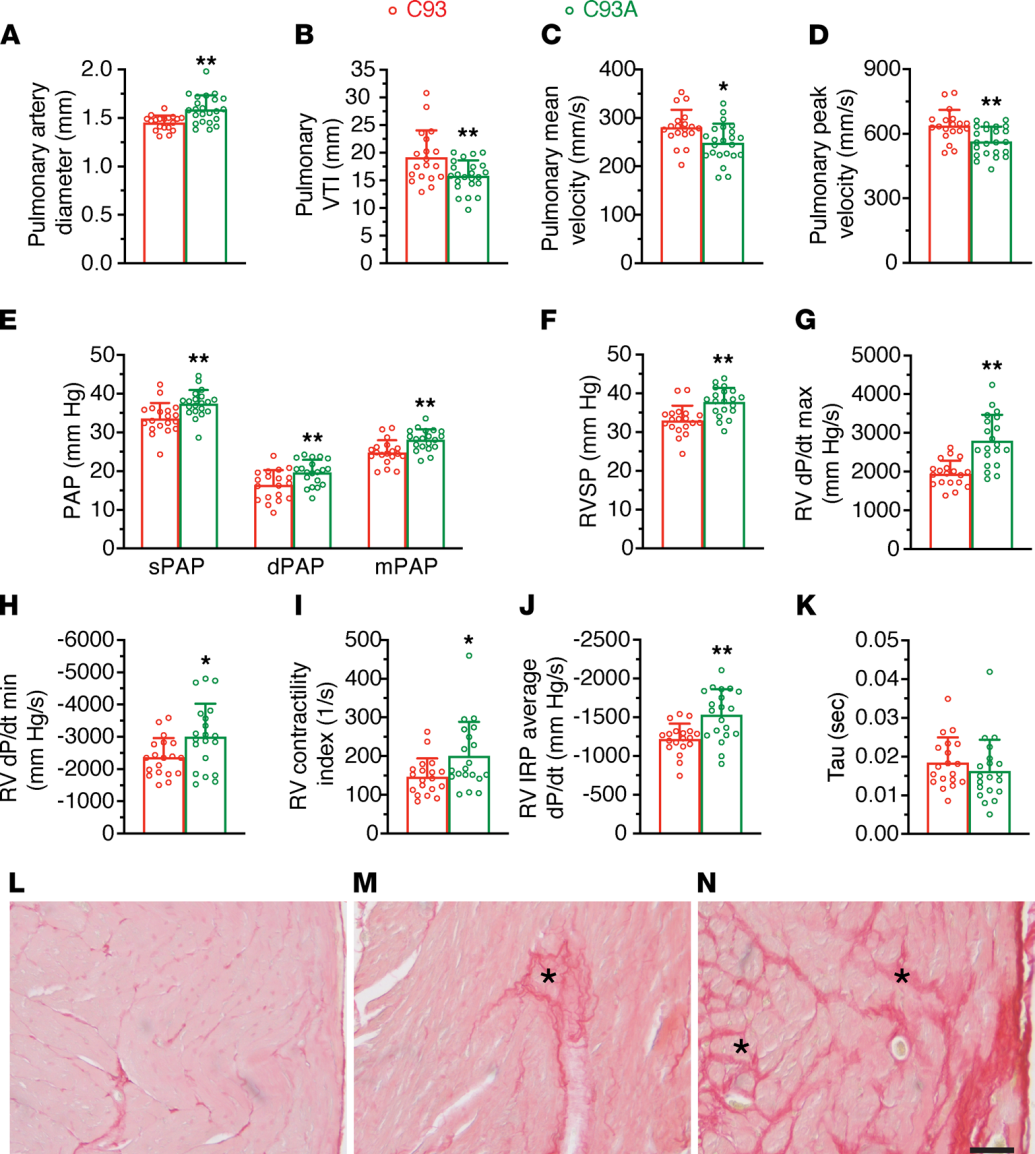
Pulmonary hypertension and right ventricular dysfunction in hypoxic C93A mice. All comparisons are between hypoxic C93A (red bar) versus C93 (green bar) mice. (**A**) Pulmonary artery diameter. (**B**) Pulmonary artery blood flow VTI. (**C**) Mean velocity of pulmonary artery blood flow. (**D**) Peak velocity of pulmonary artery blood flow. (**E**) Systolic pulmonary arterial pressure (sPAP), diastolic pulmonary arterial pressure (dPAP), and mean pulmonary arterial pressure (mPAP). (**F**) Right ventricular systolic pressure (RVSP). (**G**) Maximal rate of change in right ventricular (RV) pressure (*dP/dt* max). (**H**) Minimal rate of change in RV pressure (*dP/dt* min). (**I**) RV contractility index. (**J**) RV average *dP/dt* over isovolumic relaxation period (IRP average *dP/dt*). (**K**) Time constant of relaxation (τ). (**L**–**N**) RV fibrosis in young mice housed in 10% O_2_ for 4 weeks, visualized by Picrosirius red staining. C93 lacking fibrosis (**L**, representative of 3 tested), C93A displaying developing fibrotic areas (**M**, representative of 4 of 5), and C93A with fibrosis (**N**, observed in 1 of 5). * indicates fibrotic areas; scale bar: 50 μm. For all quantitative panels, data are presented as mean ± SD. Young mice exposed to 10% O_2_ for 4 weeks: for panels **A**–**D**, *n* = 19 C93, 4.9 ± 1.3 months of age, and *n* = 23 C93A, 4.4 ± 1.1 months of age); for panels **E**–**K**, *n* = 19 C93 mice (4.9 ± 1.3 months of age) and *n* = 20 C93A mice (4.3 ± 1.1 months of age). Differences were assessed using Student’s *t* test (2 tailed). *P < 0.05, **P < 0.01 C93A vs. C93.

Patients with chronic hypoxia-induced pulmonary hypertension have low SNO-Hb, and their RBCs show impaired vasodilatory responses in vitro (36). We therefore exposed C93A mice to chronic hypoxia. Based on our previous study demonstrating that mutant mice survived acute exposure to 10% hypoxia but succumbed quickly at 5% O_2_ (7), we housed young mice at 10% O_2_ for 4 weeks (all mice survived) and examined cardiac function and pulmonary artery pressure using echocardiography and invasive catheterization, respectively. Pulmonary artery diameter was significantly larger in C93A mice after chronic hypoxia (Figure 3A), and pulmonary VTI was diminished (Figure 3B). The mean and peak velocity of blood ejection from the right heart were also reduced in chronically hypoxic C93A mice, consistent with right heart dysfunction subsequent to pulmonary arterial hypertension (Figure 3, C and D). Indeed, systolic, diastolic, and mean pulmonary artery pressures were all significantly elevated in C93A mice versus control mice (Figure 3E). Furthermore, right ventricular systolic pressure was significantly higher in C93A mice than in C93 (Figure 3F). Analysis of pressure-time curves revealed that right ventricle *dP/dt* max, *dP/dt* min, contractility index, and average *dP/dt* over IRP (IRP average *dP/dt*) were all significantly elevated in C93A mice (Figure 3, G–J). However, the time constant of relaxation (τ) did not differ (Figure 3K). The right ventricles of C93A mice showed evidence for increased fibrosis compared with C93 controls (Figure 3, L–N). Overall, these changes are indicative of pulmonary arterial hypertension with right ventricular dysfunction.

As a confirmatory measure of the effects of chronic hypoxia, we compared young mutant C93A mice under normoxia with young C93A mice housed under 10% O_2_ for 4 weeks (using data shown in Figures 2 and 3 and Supplemental Figure 2). Pulmonary artery diameter increased in young C93A mice after chronic hypoxia (Supplemental Figure 3A). Pulmonary artery blood flow VTI, mean blood velocity, and peak blood velocity all were significantly reduced in young C93A mice (Supplemental Figure 3, B–D). Further, systolic, diastolic, and mean pulmonary artery pressures were increased in young C93A mice versus controls (Supplemental Figure 3, E–G), and right ventricular pressures, *dP/dt* max, *dP/dt* min, and average *dP/dt* over the IRP were also increased (Supplemental Figure 3, H–L). Finally, there was significant hypertrophy of the right ventricle compared with the left ventricle and of the total heart compared with body weight (Supplemental Figure 4, A–D). Thus, pulmonary hypertension with cor pulmonale is induced by hypoxia independently of age in C93A mice.

### Left heart function with aging and chronic hypoxia.

While left ventricular ejection fraction and fractional shortening were normal at baseline in young adult C93A animals (Supplemental Table 1), these functional measures were reduced in aged animals (Supplemental Figure 5, A and B). Further, chronic hypoxia led to modestly reduced left ventricular ejection fraction and fractional shortening in young C93A mice versus control C93 mice (Supplemental Figure 5, C and D; and Supplemental Figure 6, A and B), accompanied by increases in left ventricular end-systolic and -diastolic diameters and volumes (Supplemental Figure 5, E–H; and Supplemental Figure 6, C–F). Parameters that did not show differences in young C93A mice between normoxia and chronic 10% O_2_ included left ventricular end-systolic volume, inner diameter at systole, ventricular end-diastolic volume, and inner diameter at diastole (Supplemental Figure 6, C–F).

### Increased mortality in the absence of βCys93 under chronic hypoxia.

**Figure 4:**
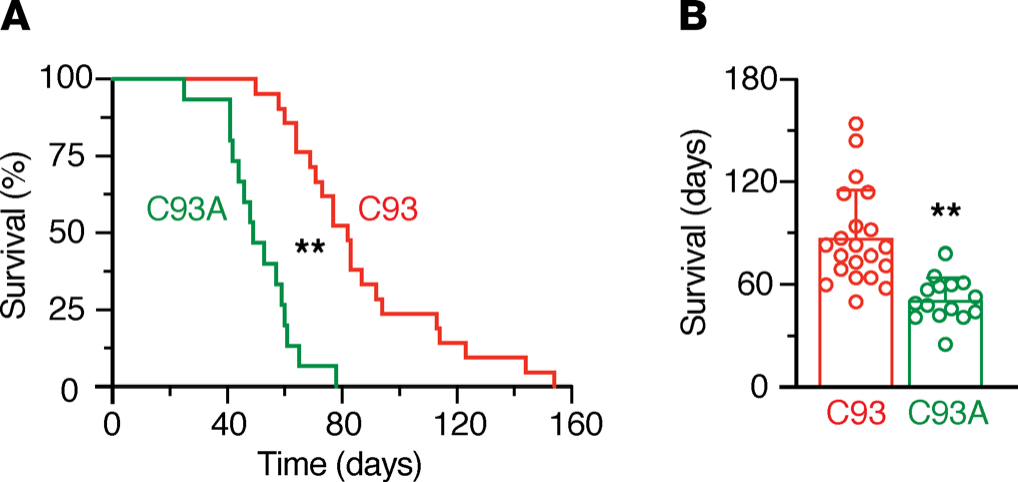
Increased mortality during chronic hypoxia in C93A mutant mice. (**A**) C93 and C93A mice were housed under 10% O_2_, and survival was assessed daily. The percentage of mice surviving each day is shown in a Kaplan-Meier plot. Curves were compared using log-rank (Mantel-Cox) and Gehan-Breslow-Wilcoxon test. **P < 0.01 C93A (*n* = 15, 7.6 ± 0.5 months of age) vs. C93 (*n* = 21, 7.8 ± 0.8 months of age). (**B**) Average days of survival at 10% O_2_, plotted as mean ± SD, with individual data points shown. **P < 0.01 C93A vs. C93 by Student’s *t* test (2 tailed).

We assessed survival of mice housed chronically under 10% O_2_. The C93 wild-type mice all survived through 50 days but were all dead by 154 days, with a mean survival time of 87 days (Figure 4, A and B). In contrast, C93A mice died much sooner, with the first mouse dying at 25 days and the last at 78 days, with a mean survival time of 51 days (Figure 4, A and B). This is consistent with mice lacking ability to mediate hypoxic vasodilation of peripheral and pulmonary vasculature.

## Discussion

The integrated response to systemic hypoxia is characterized by peripheral vasodilation, pulmonary vasoconstriction, and a central drive to breathe that is designed to restore tissue oxygenation. Here, we show that SNO derived from RBCs plays a central role in this coordinated physiology. First, we show that the ability of RBCs to induce hypoxic vasodilation in bioassays in vitro and in peripheral vessels in vivo is significantly reduced when Hb is unable to dispense SNO under hypoxia. Second, we show that SNO-Hb–deficient mice have pulmonary hypertensive changes at baseline and hallmark changes of pulmonary hypertension with chronic hypoxia. Prior work has shown that C93A mutant animals also exhibit a defect in drive to breathe (19). Collectively, these experiments provide physiological support for the model in which RBCs act as O_2_ sensors and O_2_-responsive, SNO-based vasodilators of peripheral and pulmonary vasculature. RBCs unable to liberate SNO from Hb βCys93 cannot effectively enter tissues, creating the sequelae of chronic hypoxia (ref. 7; and herein). Nature recapitulates these experiments in native Tibetans who compensate for hypobaric hypoxia through increases in RBC SNO that elevate tissue blood flow (37), while patients with pulmonary hypertension and hypoxemia exhibit losses of RBC SNO and RBC-mediated vasodilation (36). Under similar conditions herein, animals lacking Hb βCys93 die with signs of cor pulmonale.

Our demonstration of Hb βCys93-dependent hypoxic vasodilation of aortic rings in in vitro bioassays is in agreement with prior studies (7, 27) but in contrast to a recent report that found no difference between C93 and C93A RBCs loaded with SNO (22). In that report, isolated RBCs were pharmacologically treated with very high concentrations of NO donors (22), which results in high oxidation to met-Hb and SNO loading of sites other than Hb βCys93 (28) (including glutathione and sites in Hb itself). These conditions lead to vasodilation that is independent of hypoxia (i.e., artifactual vasodilation in room air) since neither met-Hb nor SNO sites other than HbβCys93 exhibit allosteric coupling to Hb oxygenation state (9, 13, 15, 25, 38). By carefully titrating the SNO donor, we were able to selectively target βCys93 to minimize met-Hb accumulation. Notably, our newly optimized conditions for mouse RBCs are quite different from those used for physiological loading of human RBCs (15), where vasodilation is entirely SNO-Hb mediated (27, 29, 38). Nonetheless, we demonstrate reduced hypoxic vasodilation by CysNO-treated mutant RBCs compared with control RBCs, reflecting differences in SNO-Hb levels, whereas residual vasodilation by C93A RBCs likely reflects alternative SNOs that are generated by CysNO, including fetal SNO-Hb (i.e., SNO-γCys93; refs. 25, 29), as well as mediators such as ATP, which are important in mouse (but not human) vasodilation in vitro (27, 35, 39).

As an in vivo correlate of RBC-mediated hypoxic vasodilation, we measured vasodilation after temporary vaso-occlusion. Upon release of occlusion, blood flow rapidly increases above the basal rate as tissue microvasculature vasodilates to maximize blood flow to O_2_-deprived tissues. The loss of half of this effect in mice bearing C93A Hb demonstrates a major role for SNO released from hypoxic Hb in mediating vasodilation and highlights a reduced ability of C93A mice to respond to hypoxic episodes. This is consistent with classic experiments showing that artery diameter changes with blood O_2_ saturation, even at constant flow (40). Indeed, the diameter of the aorta increases after restoration of blood flow, and half of this effect is also absent in C93A mice. Although shear stress on endothelial cells leading to acute NO release is believed to be responsible for this effect (refs. 32, 33, 41; often referred to as FMD), this has been controversial as eNOS inhibition (42–46) only blocks approximately 50% of the response. Thus, the suitability of the human FMD measurement to indirectly measure endothelial NO release and thus predict cardiovascular health is vigorously debated (47–49).

It is important to note that aortic dilation is observed only once hyperemic flow subsides (compare Figure 1, E and H). This suggests a direct effect of SNO-Hb on the aorta, as observed in in vitro bioassays (Figure 1C), rather than an indirect response to downstream flow. In fact, immediate hyperemic flow is associated instead with aortic constriction that is likely reflective of adrenergic responses to maintain blood pressure. Altogether, our experiments reveal a previously unappreciated role for Hb-derived SNO, and for large but transient increases in blood flow driven by RBCs rather than endothelial cells, which reflects end organ metabolic requirements, not vascular health per se. This new insight of a role for RBCs in addition to endothelium may help clarify the meaning and utility of similar assays in patients.

An exciting new report for a role of RBC eNOS in control of blood pressure (34) merits comment in this regard. Whereas eNOS in RBCs contributes to the overall blood pressure–lowering effect of NO, it plays no role in FMD, as shown in the same study (34). By contrast, Hb βCys93 plays a major role in “FMD” (this report) but has no role in blood pressure control (7, 23). Furthermore, eNOS deletion from RBCs does not affect hypoxic vasodilation by RBCs in vitro (27). Thus, eNOS apparently plays a role in one situation (blood pressure control) and Hb βCys93 in another (autoregulation of blood flow).

The new work on RBC eNOS also deserves mechanistic comment. The authors of that report (34) seem to favor a role for nitrite in blood pressure lowering by RBC eNOS, but their data show otherwise. eNOS deletion from RBCs does not reduce nitrite or overall levels of NO metabolites in RBCs. Only heme-NO levels are apparently lower in eNOS^–/–^ RBCs, which is well rationalized by lower amounts of NO produced, as NO binds heme directly. In addition, eNOS generates SNOs including SNO proteins, which may lower blood pressure, and SNO levels in plasma and RBCs are altered in mice with RBC-specific eNOS deletion (34). Thus, SNOs derived from RBC eNOS mice likely contribute to the circulating SNO pool, which mediates blood pressure lowering, just as SNOs are central to all NO bioactivity (27, 35). By contrast, there is no evidence for any role played by Hb in generation of NO that lowers blood pressure or any basis for the idea that eNOS activity requires Hb to lower blood pressure. Finally, studies that have directly assessed RBC-mediated vasodilation fail to show any activity of nitrite (25, 27); to our knowledge, not a single study has shown vasodilation by nitrite added to RBCs or Hb in physiological amounts.

We have previously reported that hypoxic vasodilation by RBCs counteracts pulmonary hypertension in animals and patients (36), implicating a role for SNO-Hb in effective V/Q matching. Our new data provide genetic support for these findings by demonstrating that mice deficient in SNO-Hb were predisposed to develop pulmonary hypertension and right heart failure. In particular, C93A mice showed signs of abnormal pulmonary reactivity and right heart strain at baseline that worsened with age. Moreover, as compared with wild-type mice, pulmonary hypertension was more severe in mutant mice housed in low O_2_ environments. Chronic hypoxia may result not only in pulmonary hypertension and right heart remodeling (50) but also in left ventricular failure in patients with heart disease, as hearts are under increased stress (51). βCys93 mutation mimics coronary heart disease by impairing vasodilation (Figure 1) and blood flow (7, 20), which would explain the left ventricular dysfunction that evolves with age or chronic hypoxia.

Hypoxic phenotypes in the C93A mice have been unnecessarily controversial, with some researchers failing to identify differences from control mice based on physiology that is not linked to hypoxic regulation (e.g., systemic blood pressure) or tests that are not up to the task (e.g., lung histology as a measure of pulmonary artery pressure; ref. 23). Thus, despite the original claim that abnormalities in pulmonary hemodynamics were not evident in these mice (23), we clearly show otherwise. Also, previous work (22, 23) did not consider the dynamic nature of hypoxic responses, which are distributed across the microcirculation and quickly fade as the system adapts (e.g., Figure 1, E and H), or the effects of age, as shown here. Further, numerous compensations in C93A mice to counterbalance the loss of SNO release from Hb (25, 29, 35) were not recognized early on. These include increased fetal Hb γCys93 SNO, a shift in SNO from C93 to other Cys residues in Hb and to glutathione, and the development of coronary collateral vessels (7, 20, 23). All this aside, the C93A mice exhibit prenatal lethality (half of predicted pups are not born; ref. 7), suggesting that the most affected individuals never survive to be tested, and those that do survive show profound functional deficits in blood flow and tissue oxygenation throughout the body (7). In addition, as shown here and elsewhere (7, 20), mice lacking SNO-Hb–mediated hypoxic vasodilation develop cardiopulmonary insufficiency and die more rapidly under hypoxic stress.

We conclude that the invariant βCys93 residue in Hb regulates both pulmonary and peripheral vascular responses to hypoxia. Animals lacking hypoxia-mediated vasodilation by SNO-Hb exhibit sine qua non features of hypoxic insufficiency and die from cardiopulmonary failure. The importance of this fundamental physiology may be alternatively appreciated in terms of hypoxic signaling where HIF/pVHL (1, 52) acts at the cellular level (via O_2_-regulated hydroxylation), while Hb/SNO acts at the organ level (via O_2_-regulated *S*-nitrosylation) to integrate system-wide responses (53). Together with previous work showing essential roles for βCys93 in blood flow autoregulation (7) and in hypoxic ventilatory responses (19), our results identify SNO-βCys93 with the essential functions of the heart, lungs, and blood in O_2_ delivery.

## Methods

### Animals.

C57BL/6J mice were purchased from The Jackson Laboratory. Mice bearing human α-globin and β-globin (plus γ-globin) genes in place of the corresponding mouse genes, either with β-globin bearing the wild-type Cys93 residue (C93), or instead carrying the Cys93Ala mutation in the human β-globin gene (C93A), were obtained from Tim Townes (University of Alabama at Birmingham, Birmingham, Alabama, USA) (23). All mice used in experiments were male (except for blood collection for SNO loading and bioassay, which used both male and female mice), and mice were tested as young or aged adults, with the age range described in the figure legend for each specific experiment.

### SNO loading of human Hb in mouse RBCs.

Mouse blood was obtained from inferior vena cava of male and female C93 and C93A mice after isoflurane euthanasia. Blood (~0.7–1.0 mL per mouse) was spun at 1500*g* for 2 minutes. Equal volumes of paired C93 and C93A samples were washed twice in PBS/EDTA, pH 7.8, by gently inverting the tubes several times. RBC pellets were resuspended at 50% hematocrit in PBS/EDTA, pH 7.8. Resuspended RBCs were diluted 8-fold to 6.25% hematocrit for SNO loading, since lower RBC density led to improved loading efficiency, as will be detailed elsewhere. Equal volumes of cysteine ethyl ester in 1N HCl and 100 mM sodium nitrite in H_2_O were mixed to generate ethyl ester CysNO and immediately diluted to 2 mM in PBS/EDTA. Ethyl ester CysNO (final concentration 200 μM; pH 7.8) was gently mixed with RBCs and incubated for 5 minutes at room temperature in the dark. The RBC suspension was centrifuged and washed twice (5 minutes at room temperature, 1500*g*) with 8 mL PBS/EDTA, pH 7.1, to remove the SNO donor, and the final cell pellet was resuspended to 50% hematocrit and used immediately for bioassays. An aliquot was used for heme and SNO quantification, and under these conditions C93 RBCs contained ~10 SNO/1000 Hb tetramer (~2 SNO/1000 heme) while C93A had ~6 SNO/1000 Hb tetramer (further details of optimization will be described elsewhere).

### Aortic ring bioassay.

RBC-mediated hypoxic vasodilation of isolated aorta was performed essentially as previously described (3, 13, 15). Briefly, thoracic aorta was dissected from C57BL/6J mice and rinsed with PBS. A 3 mm ring was hung with wires connected to a Radnoti isometric transducer and placed within an organ bath containing Krebs buffer at 37°C and bubbled with gas as indicated, either 20% O_2_ or 1% O_2_ with 5% CO_2_ and nitrogen. Rings were contracted using 1 μM phenylephrine, and tensions were recorded using a PowerLab data acquisition system and LabChart 7.3 software (ADInstruments). Assays were performed using 4 organ baths in parallel. Aorta rings in the organ bath were incubated with l-NMMA (1 mM) for 10 minutes to inhibit NOS, and glutathione (100 μM) was added to the organ bath 1 minute prior to RBC addition, to serve as an SNO carrier thiol between RBCs and aortic endothelium in the absence of blood flow; it has no effect alone (7, 54). SNO-loaded C93 and C93A humanized mouse RBCs were added to the bath such that the final concentration was 0.4% hematocrit, and change in tension was recorded over time. Changes were measured at maximal vasodilation (~2 minutes) or at 2 minutes when changes were small.

### Abdominal aorta dissection surgery.

Mice were anesthetized with 2,2,2-Tribromoethanol (0.25 mg/g i.p., MilliporeSigma) and secured in the supine position on a temperature-controlled small animal operation table. A midline incision was made to expose the abdominal cavity and the intestine carefully moved aside to reveal the abdominal aorta at the level of the left renal vein. A segment of the abdominal aorta from the inferior vena cava below the left renal vein level was separated gently, and once recording instruments were in place (see below), a 7-0 silk suture was placed around the abdominal aorta in order to occlude blood flow through ligation. Mice were used immediately for measurement of hypoxia-induced flow increases.

### Postischemia induced vasodilation.

Hypoxic vasodilation was induced using a protocol inspired by measurement of FMD of brachial artery in nonhuman primates and human patients (30, 55, 56). After the abdominal aorta dissection procedure, the postsurgery mouse was transferred to a temperature-controlled small animal operation table for echocardiography. Vascular ultrasound was performed using a Vevo 770 High-Resolution Imaging System equipped with an RMV-708 55 MHz probe (VisualSonics). Short axis M-mode images of the abdominal aorta below the left renal vein but *above* the ligation site were acquired at baseline, then the ligature around the abdominal aorta was tightened to completely occlude blood flow for 5 minutes, and then the ligation was released. After ligation release, short axis M-mode images of the abdominal aorta were captured every 30 seconds for 10 minutes. Following the experiment, mice were euthanized. The end-diastolic diameter of the abdominal aorta for baseline and for each 30-second time point was determined using the Vevo system software. Dilation was calculated as (diameter_maximum_ – diameter_baseline_)/(diameter_baseline_) × 100% to yield percentage of basal. Dynamic dilation was calculated every 30 seconds after the release of the ligature, while overall dilation was calculated using the peak diameter response after ligature release.

### Measurement of reactive hyperemia.

After abdominal aorta dissection, an ultrasound blood flow probe (MA0.5PSB, Transonic Systems Inc.) was placed around the abdominal aorta *below* the preplaced 7-0 silk suture. Abdominal aortic blood flow was measured directly with a perivascular flow meter (TS420, Transonic Systems Inc.), and after a stable period of baseline blood flow recording, the abdominal aorta was ligated using the 7-0 silk suture to occlude completely blood flow for 5 minutes; then blood flow was restored upon release of the ligature. Blood flow was continuously recorded using a PowerLab data acquisition system for 10 minutes, and data were analyzed using LabChart 7.3 software (ADInstruments). After the recordings, mice were euthanized. Mean flow increase was calculated as a percentage of baseline according to (BF_maximum_ – BF_baseline_)/(BF_baseline_) × 100%, where BF_baseline_ is the baseline mean blood flow, and BF_maximum_ is the maximum mean blood flow after ligation release; systolic flow increase was calculated in the same manner using systolic BF_baseline_ and systolic BF_maximum_. The dynamic change in flow was calculated every 15 seconds for each time point, and the overall flow increase was calculated using the peak blood flow response after ligature release. Blood pressure was not measured during these assays due to concerns that the arterial pressure transducer catheter would interfere with flow measurements.

### Chronic hypoxia.

Mice were housed in standard cages within an O_2_-controlled cabinet (Model 30, Coy Laboratory Products, Inc.) supplied with 10% O_2_. The O_2_ concentration was controlled by an O_2_ controller (Coy Laboratory Products, Inc.). Cages, drinking water, and food were changed weekly; mice were checked daily. Control mice were simultaneously housed at 21% O_2_ under identical conditions. The mice were treated with 10% O_2_ for 4 weeks for evaluation of pulmonary hypertension, or chronically in the 10% O_2_ tolerance test.

### Echocardiography.

Mice were anesthetized in a small animal incubator with 2% isoflurane in room air, then transferred and secured in the supine position on a temperature-controlled small animal operation table. Transthoracic echocardiography was performed using a Vevo 3100 imaging system equipped with an MX400 probe (FUJIFILM VisualSonics); the mice were allowed to regain consciousness and were in room air during the entire procedure. Standard M-mode sampling was captured through the left ventricular short axis at the midpapillary level. Standard B-mode and pulsed-wave Doppler for the pulmonary artery were captured for artery diameter and blood flow analysis. Ejection fraction, fractional shortening, pulmonary artery diameter, blood flow velocity, and other parameters were determined using the Vevo LAB 5.5 analysis software (FUJIFILM VisualSonics).

### Right heart and pulmonary artery catheterization for hemodynamics.

Young and aged mice at baseline, or young mice after 4 weeks of 10% O_2_ hypoxia challenge, were utilized for hemodynamic assessment. Right heart and pulmonary artery catheterization and pressure measurements were obtained using the technique reported by Skuli and colleagues (57), with slight modifications. The young mice were anesthetized with 2,2,2-Tribromoethanol (MilliporeSigma) at a dose of 0.25 mg/g i.p., while aged or hypoxia-challenged mice were given half of this dose initially, then supplemental doses of 2,2,2-Tribromoethanol as needed. Mice were secured in the supine position on a temperature-controlled small animal surgical table, and respiratory support was supplied with a rodent ventilator (MiniVent 845; Harvard Apparatus) connected through the mouth into the trachea. A midline sternotomy incision was made, and a pressure transducer (SPR-1000, Millar Instruments) was inserted through a small puncture into the right ventricle for initial recordings, then later advanced to the pulmonary artery for additional recording. Pressures were recorded using a PowerLab data acquisition system, and pressure parameters were analyzed using LabChart 7.3 software. Contractility index (max *dP/dt* normalized by left ventricular pressure, to avoid the influence by ventricular afterload) was calculated using LabChart based on the concept of Mason et al. (58). After the final measurements, mice were euthanized.

### Pathology and histology.

Untreated mice at the indicated ages, or young mice housed under 10% O_2_ chronic hypoxia for 4 weeks, were euthanized and body weight was measured. Necropsy was performed, and the wet weights of the right ventricle, left ventricle plus septum, and lung were determined. All organ weight data were normalized to the body weight of the individual mouse. Hearts were fixed in 10% neutral buffered formalin, and 8-micron, paraffin-embedded sections were stained using the Picrosirius Red Kit (Polysciences, Inc.) according to the manufacturer’s protocol. Fibrosis was assessed visually.

### Statistics.

Student’s *t* test (2 tailed) was used to compare 2 groups, and 2-way ANOVA with Holm-Šídák test was used for multiple comparisons. Kaplan-Meier survival differences were tested using log-rank (Mantel-Cox) and Gehan-Breslow-Wilcoxon test. All statistics were calculated using GraphPad Prism version 8.1 for Mac (GraphPad Software). The significance level was set at *P* < 0.05.

### Study approval.

All mouse procedures were performed under an animal protocol approved by the Case Western Reserve University IACUC.

## Author contributions

RZ and JSS conceived the study; RZ, AH, ZQ, and XL developed the methodology; RZ, AH, ZQ, and XL investigated; RZ, AH, ZQ, and RTP visualized data; JSS acquired funding; RTP and JSS performed project administration; RTP and JSS supervised the project; RTP wrote the original draft; and RZ, AH, ZQ, JSS reviewed and edited the draft.

## Supplementary Material

Supplemental data

## Version 1 12/16/2021

In-Press Preview

## Version 2 02/08/2022

Electronic publication

## References

[1] Waypa GB, Schumacker PT (2010). Hypoxia-induced changes in pulmonary and systemic vascular resistance: where is the O2 sensor?. Respir Physiol Neurobiol.

[2] Doctor A, Stamler JS (2011). Nitric oxide transport in blood: a third gas in the respiratory cycle. Compr Physiol.

[3] McMahon TJ (2002). Nitric oxide in the human respiratory cycle. Nat Med.

[4] Crawford DG (1959). Oxygen lack as a possible cause of reactive hyperemia. Am J Physiol.

[5] Ross JM (1962). Autoregulation of blood flow by oxygen lack. Am J Physiol.

[7] Zhang R (2015). Hemoglobin βCys93 is essential for cardiovascular function and integrated response to hypoxia. Proc Natl Acad Sci U S A.

[8] Hess DT (2005). Protein *S*-nitrosylation: purview and parameters. Nat Rev Mol Cell Biol.

[9] Singel DJ, Stamler JS (2005). Chemical physiology of blood flow regulation by red blood cells: the role of nitric oxide and S-nitrosohemoglobin. Annu Rev Physiol.

[10] Luchsinger BP (2003). Routes to *S*-nitroso-hemoglobin formation with heme redox and preferential reactivity in the β subunits. Proc Natl Acad Sci U S A.

[11] Salgado MT (2011). A new paramagnetic intermediate formed during the reaction of nitrite with deoxyhemoglobin. J Am Chem Soc.

[12] Gow AJ (1999). The oxyhemoglobin reaction of nitric oxide. Proc Natl Acad Sci U S A.

[13] Jia L (1996). S-nitrosohaemoglobin: a dynamic activity of blood involved in vascular control. Nature.

[14] Doctor A (2005). Hemoglobin conformation couples erythrocyte *S*-nitrosothiol content to O_2_ gradients. Proc Natl Acad Sci U S A.

[15] Pawloski JR (2001). Export by red blood cells of nitric oxide bioactivity. Nature.

[16] Gow AJ, Stamler JS (1998). Reactions between nitric oxide and haemoglobin under physiological conditions. Nature.

[17] Reynolds JD (2007). S-nitrosohemoglobin deficiency: a mechanism for loss of physiological activity in banked blood. Proc Natl Acad Sci U S A.

[18] Stamler JS (2008). SNO-hemoglobin and hypoxic vasodilation. Nat Med.

[19] Gaston B (2014). Essential role of hemoglobin beta-93-cysteine in posthypoxia facilitation of breathing in conscious mice. J Appl Physiol (1985).

[20] Zhang R (2016). Hemoglobin *S*-nitrosylation plays an essential role in cardioprotection. J Clin Invest.

[21] Gladwin MT, Schechter AN (2004). NO contest: nitrite versus S-nitroso-hemoglobin. Circ Res.

[22] Sun CW (2019). Hemoglobin β93 cysteine is not required for export of nitric oxide bioactivity from the red blood cell. Circulation.

[23] Isbell TS (2008). SNO-hemoglobin is not essential for red blood cell-dependent hypoxic vasodilation. Nat Med.

[24] Palmer LA (2008). SNO-hemoglobin and hypoxic vasodilation. Nat Med.

[25] Premont RT (2021). Red blood cell-mediated S-nitrosohemoglobin-dependent vasodilation: lessons learned from a β-globin Cys93 knock-in mouse. Antioxid Redox Signal.

[26] Dunham-Snary KJ (2017). Hypoxic pulmonary vasoconstriction: from molecular mechanisms to medicine. Chest.

[27] Diesen DL (2008). Hypoxic vasodilation by red blood cells: evidence for an s-nitrosothiol-based signal. Circ Res.

[28] Reynolds JD (2019). Regarding article, “Hemoglobin beta93 cysteine is not required for export of nitric oxide bioactivity from the red blood cell”. Circulation.

[29] Premont RT, Stamler JS (2020). Essential role of hemoglobin βCys93 in cardiovascular physiology. Physiology (Bethesda).

[30] Celermajer DS (1994). Endothelium-dependent dilation in the systemic arteries of asymptomatic subjects relates to coronary risk factors and their interaction. J Am Coll Cardiol.

[31] Rosenberry R, Nelson MD (2020). Reactive hyperemia: a review of methods, mechanisms, and considerations. Am J Physiol Regul Integr Comp Physiol.

[32] Chistiakov DA (2017). Effects of shear stress on endothelial cells: go with the flow. Acta Physiol (Oxf).

[33] Hsieh H-J (2014). Shear-induced endothelial mechanotransduction: the interplay between reactive oxygen species (ROS) and nitric oxide (NO) and the pathophysiological implications. J Biomed Sci.

[34] Leo F (2021). Red blood cell and endothelial eNOS independently regulate circulating nitric oxide metabolites and blood pressure. Circulation.

[35] Premont RT (2020). Role of nitric oxide carried by hemoglobin in cardiovascular physiology: developments on a three-gas respiratory cycle. Circ Res.

[36] McMahon TJ (2005). A nitric oxide processing defect of red blood cells created by hypoxia: deficiency of S-nitrosohemoglobin in pulmonary hypertension. Proc Natl Acad Sci U S A.

[37] Beall CM (2012). Nitric oxide in adaptation to altitude. Free Radic Biol Med.

[38] McMahon TJ (2000). Functional coupling of oxygen binding and vasoactivity in S-nitrosohemoglobin. J Biol Chem.

[39] Ellsworth ML (2016). Role of erythrocyte-released ATP in the regulation of microvascular oxygen supply in skeletal muscle. Acta Physiologica.

[40] Carrier O (1964). Role of oxygen in autoregulation of blood flow in isolated vessels. Am J Physiol.

[41] Duffy SJ (1999). Contribution of vasodilator prostanoids and nitric oxide to resting flow, metabolic vasodilation, and flow-mediated dilation in human coronary circulation. Circulation.

[42] Celermajer DS (2005). Brachial artery FMD with 5-minute distal cuff occlusion--a useful pathophysiological test after all!. J Appl Physiol (1985).

[43] Green D (2005). Point: Flow-mediated dilation does reflect nitric oxide-mediated endothelial function. J Appl Physiol (1985).

[44] Kannenkeril D (2021). Dependency of flow mediated vasodilatation from basal nitric oxide activity. Clin Physiol Funct Imaging.

[45] Linke A (2005). Flow-mediated vasodilation partially reflects nitric oxide-mediated endothelial function. J Appl Physiol (1985).

[46] Tschakovsky ME, Pyke KE (2005). Counterpoint: flow-mediated dilation does not reflect nitric oxide-mediated endothelial function. J Appl Physiol (1985).

[47] Raff U (2010). Nitric oxide and reactive hyperemia: role of location and duration of ischemia. Am J Hypertens.

[48] Atkinson G, Batterham AM (2014). When will the most important confounder of percentage flow-mediated dilation be reported and adjusted for at the study level?. Int J Cardiol.

[49] Tremblay JC, Pyke KE (2017). Flow-mediated dilation stimulated by sustained increases in shear stress: a useful tool for assessing endothelial function in humans?. Am J Physiol Heart Circ Physiol.

[51] Parati G (2018). Clinical recommendations for high altitude exposure of individuals with pre-existing cardiovascular conditions: ajoint statement by the European Society of Cardiology, the Council on Hypertension of the European Society of Cardiology, the European Society of Hypertension, the International Society of Mountain Medicine, the Italian Society of Hypertension and the Italian Society of Mountain Medicine. Eur Heart J.

[52] Wilson JW (2020). Oxygen-sensing mechanisms in cells. FEBS J.

[53] Gaston B (2020). Voltage-gated potassium channel proteins and stereoselective *S*-nitroso-l-cysteine signaling. JCI Insight.

[54] Stamler JS (1997). Blood flow regulation by *S*-nitrosohemoglobin in the physiological oxygen gradient. Science.

[55] Liuni A (2010). Observations of time-based measures of flow-mediated dilation of forearm conduit arteries: implications for the accurate assessment of endothelial function. Am J Physiol Heart Circ Physiol.

[56] Zhang X (2011). Rhesus macaques develop metabolic syndrome with reversible vascular dysfunction responsive to pioglitazone. Circulation.

[57] Skuli N (2009). Endothelial deletion of hypoxia-inducible factor-2alpha (HIF-2alpha) alters vascular function and tumor angiogenesis. Blood.

[58] Mason DT (1971). Assessment of cardiac contractility. Circulation.

