# Supplementary material for: Hypoxic vasodilatory defect and pulmonary hypertension in mice lacking hemoglobin β-cysteine93 S-nitrosylation
Source: JCI Insight. 2022 Feb 8;7(3):e155234. doi: 10.1172/jci.insight.155234 (PMC8855790; doi:10.1172/jci.insight.155234)
Supplement: Supplemental data [file jciinsight-7-155234-s280.pdf]

Hypoxic Vasodilatory Defect and Pulmonary Hypertension in Mice Lacking Hemoglobin β-Cysteine93 S-nitrosylation

Rongli Zhang, Alfred Hausladen, Zhaoxia Qian, Xudong Liao, Richard T. Premont, and Jonathan S. Stamler

Supplemental Information in this file:
Supplemental Figures 1-6
Supplemental Table 1

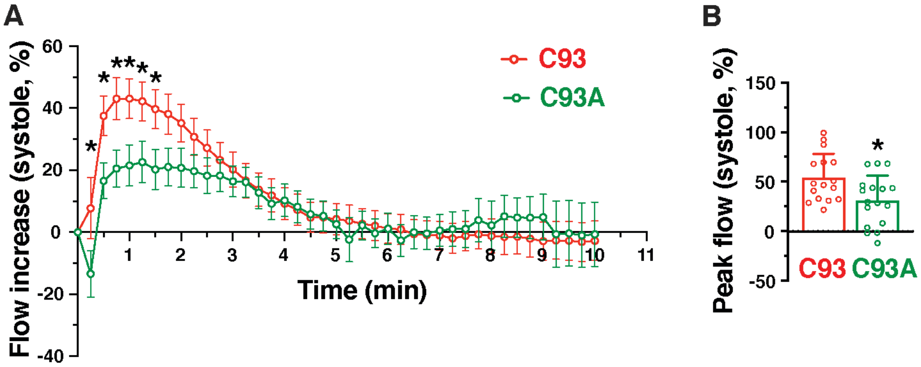

Supplemental Figure 1. **Hyperemic flow increase at systole**. (A) Systolic blood flow increase measured by ultrasound probe at the abdominal aorta in vivo after release of aortic ligature. Data are shown as mean ± SEM. n=16 C93 (3.8 ± 0.9 months of age) or n=17 C93A (3.8 ± 0.7 months of age). * p <0.05 vs. C93 by two-way ANOVA. (B) The peak systolic flow increase, calculated using the peak response from each mouse. Data are shown as mean ± SD. * p <0.05 vs. C93 by Student's t test (two-tailed).

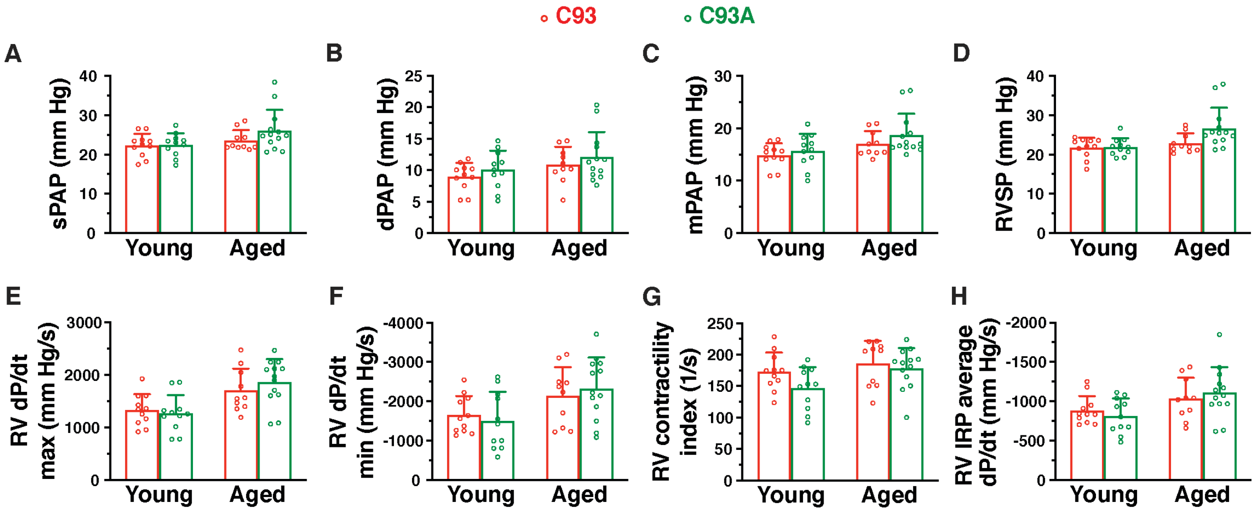

Supplemental Figure 2. **Pulmonary hemodynamics in young and aged C93A mice**. (A) Systolic pulmonary arterial pressure (sPAP) in young and in aged C93A vs C93 mice. (B) Diastolic pulmonary arterial pressure (dPAP) in young and in aged C93A vs C93 mice. (C) Mean pulmonary arterial pressure (mPAP) in young and in aged C93A vs C93 mice. (D) Right ventricular systolic pressure (RVSP) in young and in aged C93A vs C93 mice. (E) Maximal rate of change in right ventricular pressure (dP/dt max), the steepest slope during the upstroke of the pressure curve, in young and in aged C93A vs C93 mice. (F) Minimal rate of change in right ventricular pressure (RV dP/dt min), the steepest slope during the downstroke of the pressure curve, in young and in aged C93A vs C93 mice. (G) Right ventricular contractility index in young and in aged C93A vs C93 mice. (H) Right ventricular average dP/dt over isovolumic relaxation period (RV IRP average dP/dt) in young and in aged C93A vs C93 mice. For all panels, data are presented as mean ± SD, and young mice (n = 11 C93, 3.9 ± 1.0 months of age and n = 11 C93A, 3.5 ± 0.8 months of age) and aged mice (n = 10 C93, 21.7 ± 2.6 months of age and n = 13 C93A, 21.9 ± 2.2 months of age) were assessed.

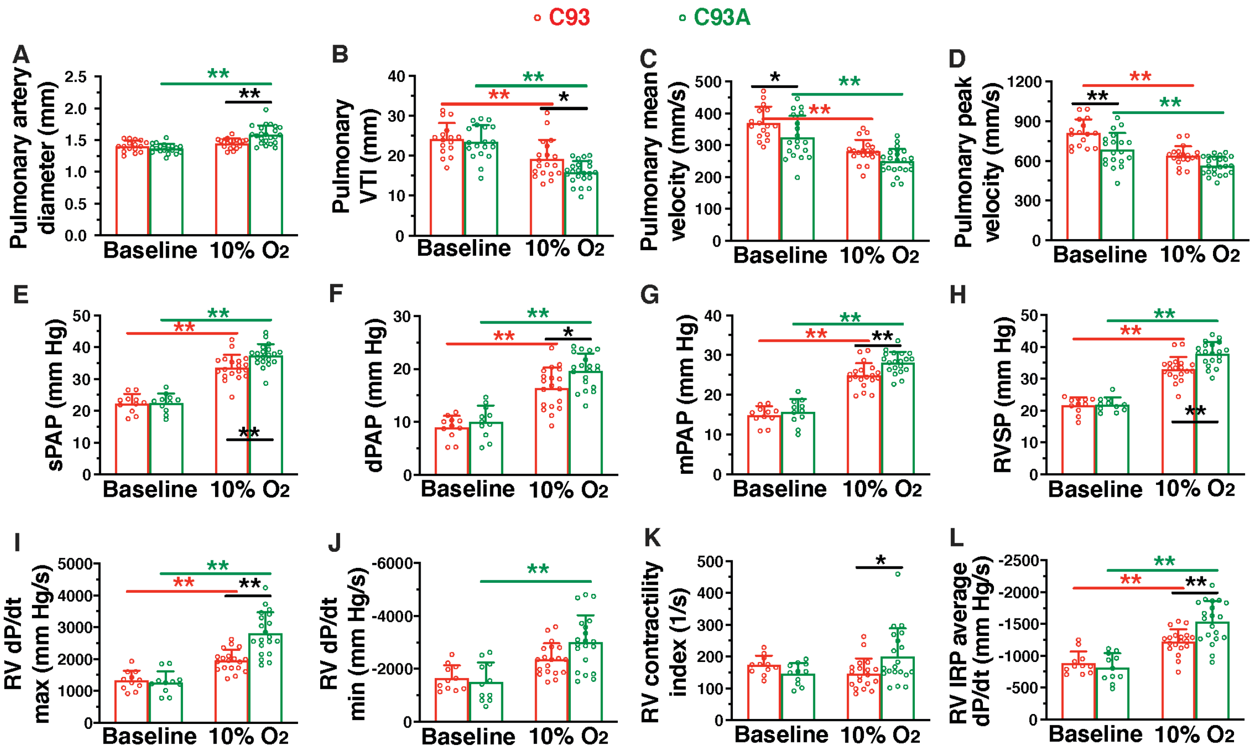

Supplemental Figure 3. Effect of chronic hypoxia on right heart function and hemodynamics in young mice housed under normoxia (baseline) or 10% O_2 for 4 weeks. (A) Pulmonary artery diameter in young C93A vs C93 mice, under normoxia (baseline) or 10% O_2 for 4 weeks. (B) Pulmonary artery blood flow velocity-time integral (VTI). (C) Mean velocity of pulmonary artery blood flow. (D) Peak velocity of pulmonary artery blood flow. (E) Systolic pulmonary arterial pressure (sPAP). (F) Diastolic pulmonary arterial pressure (dPAP). (G) Mean pulmonary arterial pressure (mPAP). (H) Right ventricular systolic pressure (RVSP). (I) Maximal rate of change in right ventricular pressure (RV dP/dt max). (J) Minimal rate of change in right ventricular pressure (RV dP/dt min). (K) Right ventricular contractility index. (L) Right ventricular average dP/dt over the isovolumic relaxation period (RV IRP average dP/dt). Data are replotted from young vs old C93 and C93A mice in normoxia (**Figure 2, Supplemental Figure 2**) and from young C93 and C93A mice in chronic hypoxia (4 weeks at 10% O_2, **Figure 3**) to highlight differences between normoxia and hypoxia in young mice. Corresponding animal group sizes and age ranges are given in the main figure legends. For all panels, data are presented as mean ± SD, * p<0.05, ** p<0.01 C93A vs. C93 by two-way ANOVA.

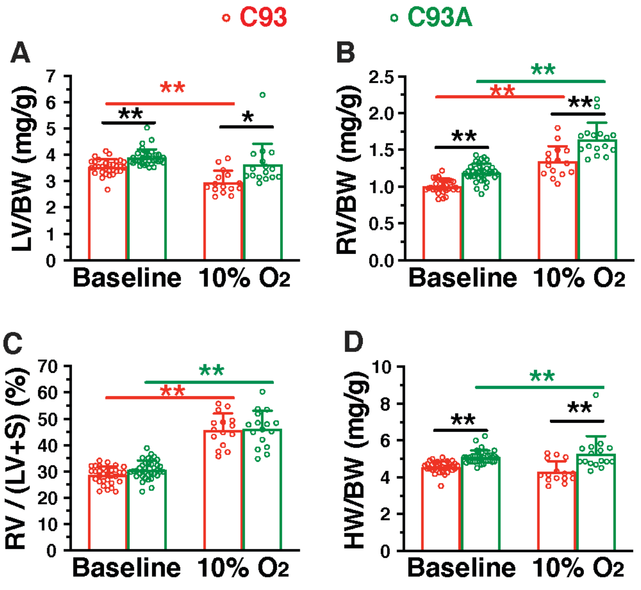

Supplemental Figure 4. Effect of chronic hypoxia on cardiac hypertrophy in young mice housed under normoxia (baseline) or 10% O_2 for 4 weeks. (A) Left ventricle (LV) to body weight (BW) ratio. (B) Right ventricle (RV) to body weight (BW) ratio. (C) Right ventricle (RV) to left ventricle + septum weight (LV+S) ratio. (D) Total heart weight (HW) to body weight (BW) ratio. Data from young C93 and C93A mice in normoxia are replotted from **Figure 2** to highlight differences between normoxia and hypoxia in young mice, and corresponding animal group sizes and age ranges are given in that figure legend. For hypoxic mice, n = 15 C93 (5.2 ± 1.3 months of age) and n = 16 C93A (3.9 ± 0.7 months of age) were used. For all panels, data are presented as mean ± SD, * $p<0.05$, ** $p<0.01$ C93A vs. C93 by two-way ANOVA.

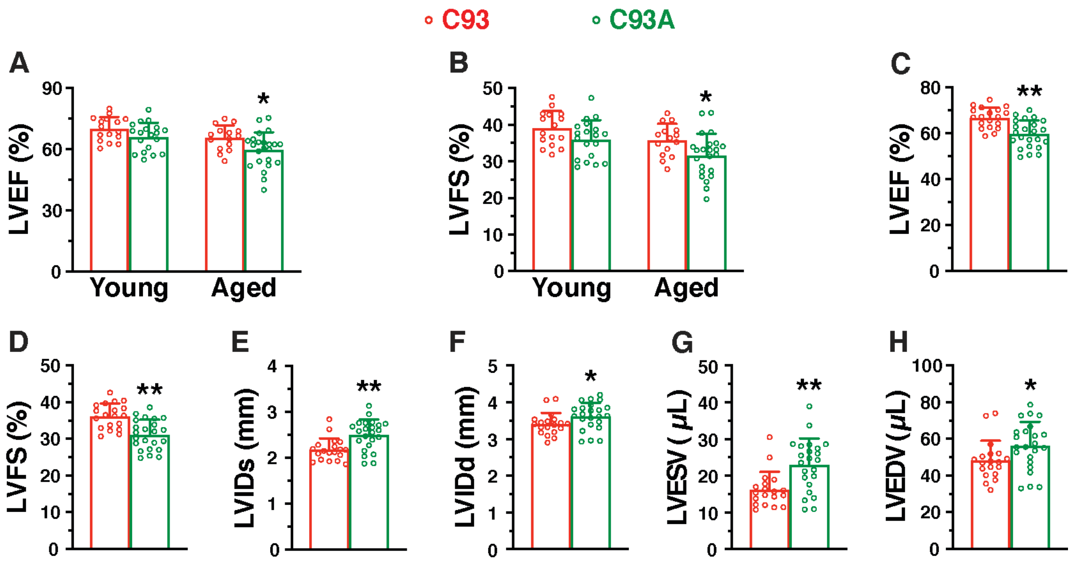

Supplemental Figure 5. Left ventricular dysfunction with age and chronic hypoxia in C93A mice. (**A**) Left ventricular ejection fraction (LVEF) in young and in aged C93A vs C93 mice. (**B**) Left ventricular fractional shortening (LVFS) in young and in aged C93A vs C93 mice. (**C**) Left ventricular ejection fraction (LVEF) in young hypoxic C93A vs C93 mice. (**D**) Left ventricular fractional shortening (LVFS) in young hypoxic C93A vs C93 mice. (**E**) Left ventricular end-systolic internal diameter (LVIDs) in young hypoxic C93A vs C93 mice. (**F**) Left ventricular end-diastolic internal diameter (LVIDd) in young hypoxic C93A vs C93 mice. (**G**) Left ventricular end-systolic volume (LVESV) in young hypoxic C93A vs C93 mice. (**H**) Left ventricular end-diastolic volume (LVEDV) in young hypoxic C93A vs C93 mice. For all panels, data are presented as mean ± SD. In panels A and B, young mice (n = 16 C93, 3.8 ± 1.3 months of age and n = 19 C93A, 3.0 ± 0.8 months of age) and aged mice (n = 15 C93, 20.9 ± 1.6 months of age and n = 23 C93A, 21.8 ± 1.2 months of age) were assessed. In panels C-H, young mice exposed to 10% O_2 for 4 weeks were assessed, n = 19 C93 (4.9 ± 1.3 months of age) and n = 23 C93A (4.4 ± 1.1 months of age). Differences were assessed using Student's *t* test (two-tailed). * $p<0.05$, ** $p<0.01$ C93A vs. C93.

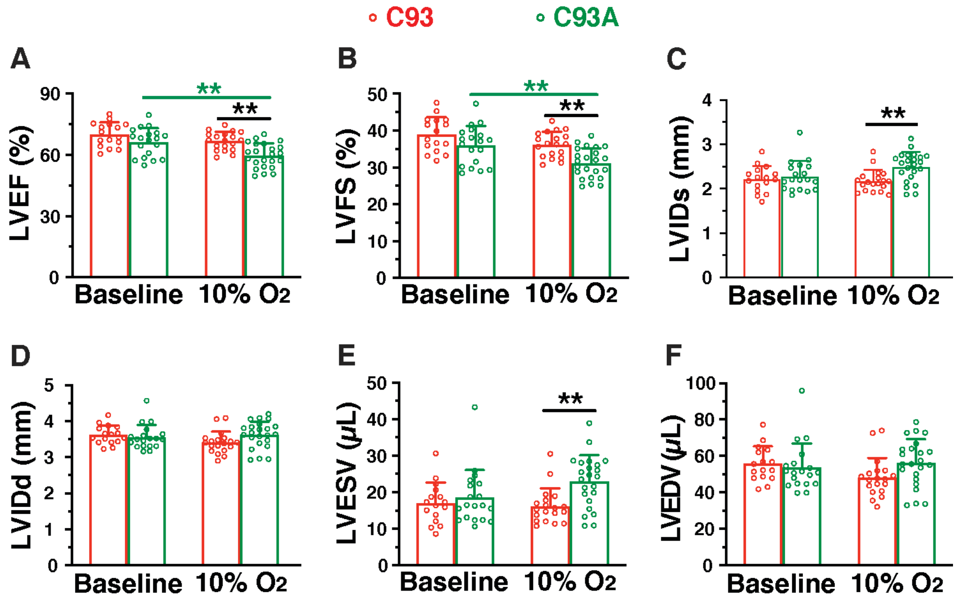

Supplemental Figure 6. Effect of chronic hypoxia on left heart function and hemodynamics in young mice. (A) Left ventricular ejection fraction (LVEF) in young C93A vs C93 mice, under normoxia (baseline) or 10% O_2 for 4 weeks. (B) Left ventricular fractional shortening (LVFS) in young C93A vs C93 mice, under normoxia (baseline) or 10% O_2 for 4 weeks. (C) Left ventricular internal diameter at systole (LVIDs) in young C93A vs C93 mice, under normoxia (baseline) or 10% O_2 for 4 weeks. (D) Left ventricular internal diameter at diastole (LVIDd) in young C93A vs C93 mice, under normoxia (baseline) or 10% O_2 for 4 weeks. (E) Left ventricular end-systolic volume (LVESV) in young C93A vs C93 mice, under normoxia (baseline) or 10% O_2 for 4 weeks. (F) Left ventricular end-diastolic volume (LVEDV) in young C93A vs C93 mice, under normoxia (baseline) or 10% O_2 for 4 weeks. Some data are replotted from **Supplemental Figure 5**, from young and old C93 and C93A mice in normoxia and young C93 and C93A mice in chronic hypoxia (4 weeks at 10% O_2), to highlight differences between normoxia and hypoxia in young mice. For panels (C)-(F), normoxia baseline groups correspond to those used in **Supplemental Figure 5A,B**. Corresponding animal group sizes and age ranges are given in the **Supplemental Figure 5** legend. For all panels, data are presented as mean ± SD, * p<0.05, ** p<0.01 C93A vs. C93 by two-way ANOVA.

Supplemental Table 1

Cardiac parameters at baseline in untreated young C93 and C93A mice

Hemodynamic parameters	C93	C93A
sPAP (mm Hg)	22.34 ± 2.96	22.45 ± 2.94
dPAP (mm Hg)	8.96 ± 2.18	10.06 ± 3.05
mPAP (mm Hg)	14.87 ± 2.28	15.69 ± 3.21
RVSP (mm Hg)	21.67 ± 2.56	21.89 ± 2.20
RV dP/dt max (mm Hg/s)	1332.46 ± 302.66	1266.30 ± 353.95
RV dP/dt min (mm Hg/s)	-1657.95 ± 480.26	-1507.31 ± 732.60
RV contractility index (1/s)	173.11 ± 30.01	146.61 ± 33.56
RV IRP average dP/dt (mm Hg/s)	-884.81 ± 181.61	-815.25 ± 222.41

Anesthetized young mice at baseline. Mean ± SD, n=11 for C93 and for C93A.

Echocardiographic parameters	C93	C93A
LVEF (%)	69.97 ± 5.91	66.15 ± 6.89
LVFS (%)	38.99 ± 4.73	35.99 ± 5.23
LVIDs (mm)	2.22 ± 0.29	2.28 ± 0.34
LVIDd (mm)	3.62 ± 0.26	3.56 ± 0.34
LVESV (µL)	17.04 ± 5.62	18.53 ± 7.57
LVEDV (µL)	55.70 ± 9.62	53.71 ± 13.19
Pulmonary VTI (mm)	24.15 ± 4.08	23.49 ± 4.22
Pulmonary artery diameter (mm)	1.41 ± 0.09	1.37 ± 0.07
Pulmonary mean velocity (mm/s)	369.97 ± 51.15	* 324.38 ± 67.99
Pulmonary peak velocity (mm/s)	811.19 ± 103.24	** 685.73 ± 125.91

Conscious young mice at baseline. Mean ± SD, n=16 for C93 and n=19 for C93A.
* p<0.05, ** p<0.01 C93A vs. C93 by two-way ANOVA.
